# Prevalence of vitamin D deficiency in critically ill patients and its influence on outcome: experience from a tertiary care centre in North India (an observational study)

**DOI:** 10.1186/2052-0492-1-14

**Published:** 2013-12-20

**Authors:** Afzal Azim, Armin Ahmed, Subhash Yadav, Arvind K Baronia, Mohan Gurjar, Madan M Godbole, Banani Poddar, Ratender K Singh

**Affiliations:** Department of Critical Care Medicine, Sanjay Gandhi Postgraduate Institute of Medical Sciences, Raebareli road, Lucknow, Pin-226014 India; Department of Endocrinology, Sanjay Gandhi Postgraduate Institute of Medical Sciences, Lucknow, India

**Keywords:** Vitamin D deficiency, Critically ill, Prevalence, Outcome

## Abstract

**Background:**

Limited studies are available on prevalence and severity of vitamin D deficiency in a critically ill population. To the best of our knowledge, this the first study of its kind in an Indian intensive care set-up.

**Methods:**

One hundred fifty-eight critically ill patients were prospectively enrolled for over 2 years. Demographic profile and clinical characteristics were noted. Blood sample for serum 25 (OH) D was collected on admission (4 ml). Serum 25 (OH) D was measured using radioimmunoassay kit. Vitamin D deficiency was labelled as insufficient (31–60 nmol/l), deficient (15–30 nmol/l) and undetectable (<15 nmol/l). Statistical tests used were *t* test, chi-square test and binary logistic regression.

**Results:**

Vitamin D deficiency (<60 nmol/l) was present in 127 patients (80.4%). Twenty-six patients had (20.47%) undetectable vitamin D levels. The mean vitamin D level was higher amongst survivors (43.17 + 39.22) than in non-survivors (39.72 + 29.31). Vitamin D was not significantly associated with mortality in univariate analysis. Multiple logistic regression showed admission APACHE II (*p* = 0.008), lactate (*p* = 0.013) and pre-ICU hospital stay (*p* = 0.041) as independent predictors of mortality in critically ill patients (*p* < 0.05).

**Conclusions:**

Vitamin D deficiency is highly prevalent in critically ill patients. A causal association between vitamin D deficiency and mortality was not found in our study. Larger studies are needed to understand the relationship between vitamin D deficiency and ICU outcome.

## Background

The pandemic of vitamin D deficiency even in effluent societies has pointed out the growing distance of human race from nature. Recent research has generated interest in pleiotropic actions of vitamin D all over the world.

Vitamin D receptors are found in nearly all types of immune cells [1]. Its action on innate immunity is stimulatory, while its action on adaptive immunity is mainly considered to be modulatory [2]. Various clinical studies and trials have shown correlation between vitamin D and systemic infections. Its deficiency has been associated with acute respiratory tract infections [3, 4], cardiovascular diseases and other chronic illnesses [5]. Scientists are now exploring its possible association with acute life-threatening illnesses. A large US-based study reviewing national database of hospital discharge from 1979 to 2003 showed seasonal variation in incidence of sepsis and severe sepsis paralleling the seasonal fluctuation in 25(OH) D levels [6, 7]. A recently published study showed that vitamin D deficiency prior to hospital admission predisposes for acute kidney injury in critically ill patients [8]. We conducted this study to analyse the prevalence and severity of vitamin D deficiency in a critically ill Indian population as there is paucity in Indian literature.

## Methods

It is a prospective single-centre study conducted in the department of critical care medicine of a tertiary care public referral hospital. The intensive care unit (ICU) admits both medical and surgical cases. Ethical clearance was obtained from the ethical committee of Sanjay Gandhi Postgraduate Institute of Medical Sciences before conducting the study. Informed consent was taken from next of kin of all enrolled patients. This study was conducted between May 2010 and May 2012.

All consecutive critically ill patients requiring ICU admission were screened for enrolment for the study. Exclusion criteria included age less than 18 years, pregnancy, patients on corticosteroid therapy, patients with history of malabsorption syndrome, patients with chronic kidney disease (CKD), patients on drugs interfering with vitamin D metabolism and patients already on vitamin D supplementation. A total of 158 patients (out of 480 admissions) met the inclusion criteria, and data was analysed for them.

Blood samples for laboratory measurements of serum 25 (OH) D were collected on patients' admission (4 ml), which were then immediately transferred to the endocrinology laboratory. Serum 25 (OH) D was measured using a radioimmunoassay kit technique using the 12 well gamma Counter machine (STRATEC, Birkenfeld, Germany). Till the samples were not processed, they were stored at −20°C temperature after separating the serum. Vitamin D deficiency was defined as values less than 60 nmol/l. Severity of vitamin D deficiency was labelled as insufficient if the level was between 31 and 60 nmol/l, deficient if the level was between 15 and 30 nmol/l and undetectable if the level was less than 15 nmol/l. The demographic profile (age/sex, co-morbidities), admission diagnosis, clinical characteristics and biochemical parameters of each patient were noted at the time of enrolment in the study. Severity of critical illness was assessed by admission acute physiological and chronic health evaluation (APACHE II) score and sequential organ failure assessment (SOFA) score. Levels and severity of vitamin D deficiency were compared between survivors and non-survivors and correlated with the outcome. Vitamin D was also compared to other predictors of outcome like APACHE II, SOFA and total days of mechanical ventilation. Laboratory and clinical management was done as per the treating team.

### Statistical analysis

Data was analysed using SPSS 16.0, and results were expressed as mean ± standard deviation or median (interquartile range) as appropriate. Sample size was decided based on significance level (alpha) = 0.05. Student's *t* test was used between survivors and non-survivors. Non-parametric test like chi-square test was used for comparison between two groups for ranked observation. Actual *p* values were reported, and a *p* value <0.05 was taken as significant. Multivariate analysis by binary logistic regression was used. All the study variables were considered, and the best model with five variables which gave 90.5% overall correct classification was used.

## Results and discussion

### Results

Of the 158 patients enrolled in the study, there were 97 male patients (61.39%) and 61 female patients (38.60%). Their mean age, SOFA score, APACHE II score, Vitamin D levels, average length of stay and average days of mechanical ventilation are mentioned in Table 1.

**Table 1:** **Patients demography (** ***n*** **= 158)**

Variable	Mean (±SD)
Age	48.50 ± 16.49
Sex	
Male	97
Female	61
Medical/surgical	137/21
Admission diagnosis	
Cardiac disease	5
Respiratory disease	45
Abdominal disease	50
Neurological disease	12
Tropical illness	35
Others (poisoning, snakebite, etc.)	11
Admission source	
Community	10
Hospital ward	53
Other ICUs/HDUs (including post-operative ICU)	95
Admission SOFA score	9.56 ± 4.32
Admission APACHE II score	15.35 ± 6.14
Vitamin D levels (nmol/l)	41.53 ± 34.29
Length of ICU stay (days)	16.84 ± 18.44
Days of mechanical ventilation	13.35 ± 17.01

Survivors and non-survivors were compared for demographic characteristics like age, severity of illness (SOFA and APACHE II) and admission laboratory parameters which included procalcitonin (PCT), serum albumin, arterial lactate, serum creatinine and levels of vitamin D (Table 2). Admission SOFA, APACHE II score, lactates, days of mechanical ventilation and days of pre-ICU stay were significantly higher in non-survivors on univariate analysis. There was no statistically significant difference in vitamin D levels amongst the two groups (*p* = 0.53).

**Table 2:** **Patient characteristics: survivors vs non-survivors**

Variable	Survivors	Non-survivors	***t*** value	***p*** value
	(***n*** = 75)	(***n*** = 83)		
	(Mean ± SD)	(Mean ± SD)		
Age (years)	47.01 ± 15.78	50.08 ± 17.05	1.17	0.25
SOFA score	7.96 ± 4.21	11.00 ± 3.94	4.66	0.001
APACHE II score	12.89 ± 5.39	17.50 ± 5.94	5.06	0.001
Procalcitonin (ng/ml)	14.14 ± 41.29	21.01 ± 60.35	0.82	0.41
Albumin (mg%)	2.74 ± 0.59	2.61 ± 0.59	1.41	0.16
Creatinine (mg%)	2.17 ± 2.20	2.78 ± 1.97	1.83	0.07
Lactate (mg/dl)	16.66 ± 9.72	24.31 ± 18.41	2.82	0.001
Vitamin D (nmol/l)	43.17 ± 39.22	39.72 ± 29.31	0.63	0.53
Pre-ICU hospital stay (days)	6.0 ± 6.02	10.67 ± 14.54	2.59	0.01
ICU length of stay	19.03 ± 20.98	14.90 ± 15.75	1.40	0.16
Days of MV	10.05 ± 19.55	15.99 ± 13.49	2.23	0.03

*MV* mechanical ventilation.

Vitamin D deficiency (<60 nmol/l) was present in 127 patients (80.4%). Fifty-three patients (41.73%) were in the insufficient group, 48 patients (37.79%) were in the deficient group and 26(20.47%) patients had undetectable vitamin D levels.

Factors found to be significantly associated with mortality in univariate analysis (SOFA score, APACHE II score, lactate, pre-ICU stay and days of mechanical ventilation) were subjected to binary logistic regression. APACHE II score, lactate and pre-ICU stay were found to be independently associated with mortality (Table 3).

**Table 3:** **Multivariate analysis of significant variables**

Variable	Logistic regression coefficient (beta)	Standard error of regression coefficient (beta)	Significance of beta	Odds ratio	95% CI of OR
SOFA	−0.081	0.054	0.133	0.922	0.829–1.025
APACHE II	−0.106	0.040	0.008	0.900	0.832–0.973
Pre-ICU stay	−0.061	0.030	0.041	0.941	0.887–0.998
Lactate	−0.053	0.022	0.013	0.948	0.909–0.989
Days of mechanical analysis	−0.022	0.012	0.072	0.955	0.829–1.002
Constant	4.078	.757	0.000	59.018	

*MV* mechanical ventilation, *OR* odds ratio. Degree of freedom = 1.

### Discussion

Vitamin D deficiency is highly prevalent in the general population all over the world. Prevalence of levels <50 nmol/l is reported between 36% and 57% in the USA and even higher (between 28% and 100%) in European studies depending upon the group of population tested and cut-off levels used for normal range [9]. Even Indian literature suggests vitamin D deficiency between 50% and 90% in the general population [10, 11].

Cut-off value of normal range of vitamin D remains a debatable topic, and whether levels considered normal for general population can be applied to critically ill patients remains unclear [12, 13]. In our study, we have used 60 nmol/l as the cut-off value based on the study published by Lee et al. in 2009 [14]. However, in a recent meta-analysis of prospective cohort studies, Zitterman and colleagues reported 75–87.5 nmol/l as the optimal concentration in the general population and even showed a non-linear decrease in mortality as circulating 25 (OH) D level increases [15].

Vitamin D deficiency and its relation with increased mortality in the general population are well established. Almost all chronic illnesses associated with ageing are adversely affected by vitamin D deficiency [16, 17]. A meta-analysis published in 2007 showed that ordinary dose vitamin D supplementation is associated with reduction in total mortality in the general population [18]. Melamed and colleagues reported that vitamin D <17.5 ng/l is independently associated with mortality in the general population [19]. Review of literature and clinical studies on end-stage renal disease patients have shown that vitamin D supplementation is associated with decreased mortality [20]. A recently published meta-analysis of 10 studies with a cohort of 6,853 patients concluded that higher vitamin D levels correlates with improved survival in CKD patients [21].

Vitamin D deficiency in critical illnesses can be multifactorial and can influence the sepsis cascade through several mechanisms. These mechanisms may include immune modulation, suppression of exaggerated inflammatory response, enhanced phagocytosis, chemotaxis, increased production of antimicrobial peptide cathelicidin, and calcium and glucose homeostasis [22, 23].

Deficiency of vitamin D in a critically ill population has also been studied, though to a lesser extent. Its association with mortality in this population subgroup remains unclear. Studies have shown a vitamin D deficiency of more than 90% in a critically ill population [24, 25]. Lee et al. reported undetectable levels in 17.5% of patients admitted to the ICU [14]. Data regarding vitamin D deficiency in the critically ill population in the Indian scenario is lacking. In our study, we found that 80.4% of patients were deficient (level <60 nmol/l) in vitamin D levels on admission to the intensive care unit, with 20.48% having undetectable levels (level <15 nmol/l). However, we could not find a causal relationship between vitamin D deficiency and mortality.

Cecchi et al. conducted a single-centre study on 170 patients, out of which 92 were severe sepsis/septic shock patients and 72 were trauma patients [26]. Vitamin D levels were significantly lower in the septic group but did not prove to be a mortality predictor on multivariate analysis. In contrast, a few studies have shown a significant association between vitamin D levels and mortality in critically ill patients [27, 28]. Mathews et al. studied the effect of vitamin D deficiency in 258 surgical ICU patients and reported an inverse relation between vitamin D deficiency and length of ICU stay, cost of therapy and mortality [29]. Braun et al. studied pre-admission 25(OH) D levels drawn 7 to 365 days prior to ICU admission in a large cohort of 2,399 patients [30]. They reported vitamin D as a strong predictor of ICU mortality. It is postulated that acute fluid shifts seen in severe sepsis and septic shock can influence the assessment of correct vitamin D levels [31]. Venkatesh et al. pointed out the wide fluctuation in vitamin D level occurring in critically ill patients for hours [32]. This could be one of the causes of different findings in various studies.

Larger studies are needed to understand the role of vitamin D level in critical illness, to define supplementation strategy, in monitoring frequency and in effect of replacement on outcome. Moreover, redesigning of ICU set-ups in such a way that all patients get adequate sunlight might be a topic of debate in modern medicine.

## Conclusions

The authors conclude that this study shows high prevalence of vitamin D deficiency in critical care settings even in tropical countries like India. We also found that APACHE II, lactate and pre-ICU hospital stay are independent predictors of mortality in critically ill patients. We could not find a causal association between vitamin D deficiency and mortality.
